# Psychosis as alzheimer disease debut: a case report

**DOI:** 10.1192/j.eurpsy.2022.2293

**Published:** 2022-09-01

**Authors:** C. Vilella Martín, P. García Vázquez, Y. Barrera García, P. Fernández Perea, M.Á. Heredero Sanz, A. Serrano García, R. Gómez Martínez, C. Franch Pato

**Affiliations:** Complejo Asistencial Universitario de León, Psychiatry, LEÓN, Spain

**Keywords:** Alzheimer’s disease, Psychosis

## Abstract

**Introduction:**

Psychosis in Alzheimer’s disease has an incidence of ˜ 10% per year. Recent work has focused on the presence of psychosis in people with mild cognitive impairment, as a risk factor for the development of Alzheimer’s disease.

**Objectives:**

To Study a case of Alzheimer’s disease presenting psychotic symptoms

**Methods:**

Retrospective review of clinical records and complementary test, including psychiatry, electrophysiology and neurology.

**Results:**

A 40-year-old goes to the emergency room due to hetero-aggression at home. He says that his father steals his money and prostitues have been hired in his house. The patient is oriented, partially collaborative and approachable. Psychomotor restlessness is observed. He has self-referral delusions, auditory hallucinations and insomnia. Provisional diagnosis of acute psychotic episode made and low dose risperidone was prescribed. During his stay on the hospital Ward, sedation, recent memory alterations, spatio-temporal disorientation lack of initiative and disorganized behaviors appear. Risperidone is withdrawn and complementary test are performed. Imaging tests show temporal and frontal atrophy. Increased TAU protein and low levels of amyloid in CSF are found. Brain biopsy is +. His mother died of Alzheimer’s disease with 36 years-old and another affected brother with 42 yeras-old. The definitive diagnosis is Alzheimer’s disease and genetic studies are currently being carried out.

**Conclusions:**

Alzheimer’s disease can debut with psychosis. It is important to investigate family history of patients who begin with memory loss in the context of psychosis

**Disclosure:**

No significant relationships.

